# Supplementary material for: Towards a Didactic Concept for Heterogeneous Target Groups in Digital Learning Environments—First Course Implementation
Source: J Pers Med. 2022 Apr 27;12(5):696. doi: 10.3390/jpm12050696 (PMC9147950; doi:10.3390/jpm12050696)
Supplement: Supplementary file 1 [file jpm-12-00696-s001.zip › 2022-01-30-Fifth_Statement_Expert_Council_Federal_Government_En.pdf]

**5. statement of the expert council of German Federal Government on COVID-19**
On the need for evidence-based risk and health communication.

**Date of publication:** 30.01.2022

**Paraphrasing translation as an accessory of the contribution:** 08.02.2022

**Current starting point**

Most citizens want to actively contribute to end the pandemic and behave according to applicable and reasonable rules. A lack of consistency of available information, its assessments, and resulting recommendations contributes to public uncertainty, provides a target for misinformation and disinformation, undermines trust in government action, and jeopardizes the success of important health protection measures. To support individual and societal self-efficacy and risk-competent behavior, responsive, evidence-based, audience- and user-specific risk and health communication is essential. This must explain scientific evidence simply, translate it into recommendations for action, and become the reference standard and first choice for helpful and reliable information. Although authorities and ministries are currently implementing parts of this, there is no institution in Germany that implements risk and health communication coordinated according to the principles listed below.

**Building blocks of effective risk and health communication**

In a decentralized and pluralistic society like Germany's, there will always be diverse actors will inform and communicate. Within the framework of this diversity, there must be professionally sound and evidence-based health communication, which is developed and implemented by multidisciplinary and ideally enjoys a high level of trust among the population. This requires at least four building blocks that are closely intertwined.

The first building block is the **generation** of the best available knowledge. This includes structures that a) enable the pooling of medical and epidemiological information, i.e., the evaluation and interpretation of relevant studies, modeling, health-related statistics and key figures; b) carry out regular monitoring of behaviorally relevant aspects such as acceptance of measures, willingness to vaccinate, trust of the population, etc., and c) allow the monitoring of classic and social media to identify trends and misinformation. These structures should be created to allow user-centered communication based on this knowledge. The Corona pandemic has made obvious the lack of availability of important data compared to other countries, and shows how this systemically tolerated lack of data hampers scientific analysis and response to the pandemic. Overall, the lack of digitization in the healthcare system in Germany (see also the 4th statement) is a major obstacle-not least for successful communication.

The second building block is the **translation** of relevant data, statistics and key figures into user-centered and target-group-specific, comprehensible, decision- and action-relevant information formats. The goals should be education, not advertising or persuasion. persuasion ("persuading"). In the interest of informational justice, the content should be adapted to different educational backgrounds, cultural, linguistic and age-related differences and be differences and be personally relevant. The translation of data should be based on the existing large body of evidence on effective communication of scientific content, risks, and uncertainties. The targeting and content planning process Shall incorporate knowledge gained from observation of behaviorally relevant aspects and (social) media as well as participatory approaches (e.g.: Who rejects vaccinations and for what reasons? Who needs to be particularly reached with what content? Who obtains information from from which sources?). As far as possible, concrete decision-making aids should be offered, such as

checklists, simple decision trees, decision heuristics, each of which can also be made existing apps, e.g. the Corona warning app, can be made available automatically.

Today, every communication campaign is in competition with misinformation and disinformation. The task of an effective communication and information strategy is therefore also to identify, evaluate and rebut these in a professional and comprehensible manner. This information must be widely known and extremely easy to access. International networking and cooperation with other national and international health organizations is essential here. After all, like the virus, infodemics - the rapid and widespread spread of both correct and incorrect information - is a global phenomenon. Here, too, there are science-based approaches and guidelines that should be implemented.

The third component is the **dissemination** of communicative content via the multiple channels of a modern information society, from classic to social media to e- and m-health offerings. The model for the dissemination process should be modern campaigns with a wide reach and media diversity. In this context, it is again of central importance to select dissemination channels that are specific to the target group. It must be taken into account that some target groups, such as children and young people, people with a migration background, or those who are educationally disadvantaged, can be reached differently via traditional or modern media repertoires. Therefore, multipliers who are active in interpersonal communication, e.g., in vaccination counseling centers or in the real world (e.g., doctors, social workers, teachers, nurses), are also particularly relevant. It is therefore important to provide them with adequate materials and an appropriate mandate to support them in the dissemination of information and personal communication. The same applies to health offices, so that all actors are always on the same and up-to-date level of advice and information, in order to avoid contradictory information to the population. Who is considered a trustworthy source also differs according to the target group (e.g., religious functionaries instead of authorities, social environment instead of scientists). This also needs to be taken into account. Last but not least, cooperation with representatives of (scientific) journalism is needed.

The final building block is the **evaluation** of the effects achieved and, if necessary, the adjustment of the strategy. Evaluation should begin at the translation stage to test the impact of content and formats and anticipate unintended effects. Involving citizens, e.g., in focus groups or experimental studies, can increase the effectiveness of the communication as well as the trust in the communicators.

**Creating sustainable communication structures**

One of the most important lessons learned from the Corona pandemic is: fact-based and action-oriented information for the population in health crises is indispensable. The Council of Experts therefore recommends improving current communication and information services according to the principles described above. The Council of Experts further proposes that the infrastructure for risk and health communication be expanded rapidly. To this end, existing competencies should be bundled and missing ones supplemented. This multidisciplinary infrastructure should be independent. It should generate the best available knowledge and translate it for the population and the professional public, disseminate it to all relevant target groups and evaluate the effect. The Corona pandemic is just one of several collective and global health crises to which society must respond. Therefore, there is a need to establish a sustainable infrastructure to provide evidence-based, rapid, and effective information and risk and action support to the population.

**Consent in the Expert Council: 18 out of 18**
